# Incidental or Intentional? Different Brain Responses to One's Own Action Sounds in Hurdling vs. Tap Dancing

**DOI:** 10.3389/fnins.2020.00483

**Published:** 2020-05-13

**Authors:** Nina Heins, Jennifer Pomp, Daniel S. Kluger, Ima Trempler, Karen Zentgraf, Markus Raab, Ricarda I. Schubotz

**Affiliations:** ^1^Department of Psychology, University of Muenster, Münster, Germany; ^2^Otto Creutzfeldt Center for Cognitive and Behavioral Neuroscience, University of Muenster, Münster, Germany; ^3^Institute for Biomagnetism and Biosignalanalysis, University of Muenster, Muenster, Germany; ^4^Department of Movement Science and Training in Sports, Institute of Sport Sciences, Goethe University Frankfurt, Frankfurt, Germany; ^5^Department of Performance Psychology, Institute of Psychology, German Sport University Cologne, Cologne, Germany; ^6^School of Applied Sciences, London South Bank University, London, United Kingdom

**Keywords:** human action sounds, auditory action effects, action-effect association, prediction, sensory attenuation

## Abstract

Most human actions produce concomitant sounds. Action sounds can be either part of the action goal (GAS, goal-related action sounds), as for instance in tap dancing, or a mere by-product of the action (BAS, by-product action sounds), as for instance in hurdling. It is currently unclear whether these two types of action sounds—incidental or intentional—differ in their neural representation and whether the impact on the performance evaluation of an action diverges between the two. We here examined whether during the observation of tap dancing compared to hurdling, auditory information is a more important factor for positive action quality ratings. Moreover, we tested whether observation of tap dancing vs. hurdling led to stronger attenuation in primary auditory cortex, and a stronger mismatch signal when sounds do not match our expectations. We recorded individual point-light videos of newly trained participants performing tap dancing and hurdling. In the subsequent functional magnetic resonance imaging (fMRI) session, participants were presented with the videos that displayed their own actions, including corresponding action sounds, and were asked to rate the quality of their performance. Videos were either in their original form or scrambled regarding the visual modality, the auditory modality, or both. As hypothesized, behavioral results showed significantly lower rating scores in the GAS condition compared to the BAS condition when the auditory modality was scrambled. Functional MRI contrasts between BAS and GAS actions revealed higher activation of primary auditory cortex in the BAS condition, speaking in favor of stronger attenuation in GAS, as well as stronger activation of posterior superior temporal gyri and the supplementary motor area in GAS. Results suggest that the processing of self-generated action sounds depends on whether we have the intention to produce a sound with our action or not, and action sounds may be more prone to be used as sensory feedback when they are part of the explicit action goal. Our findings contribute to a better understanding of the function of action sounds for learning and controlling sound-producing actions.

## Introduction

Most actions produce sounds. On a subjective level, we would say that some of these action sounds are the proper goal of the action (*goal-related action sounds, GAS*, hereafter), for instance in musical performance, singing, and speaking; whereas others occur rather as a by-product (*by-product action sounds, BAS*), for instance when we unlock a door or write on our laptop. Although this simple observation suggests potentially different categories of sound-producing actions, it remains to be experimentally addressed whether they indeed differ on the behavioral or neural level. Some findings point toward significant differences between GAS and BAS actions. In speech and musical performance, experimentally distorted or missing action sounds result in poorer performance (Howell, 2004; Pfordresher and Beasley, 2014). Omitted sounds disrupt GAS action performance permanently (Jones and Keough, 2008; Tourville et al., 2008). In contrast, Kennel et al. (2015) investigated the influence of masked and delayed online action sounds during hurdling performance, i.e., a BAS action. Authors found an interfering effect of delayed auditory feedback persisted only for the first trial of performance and vanished afterwards. Moreover, the complete masking of auditory feedback did not even transiently affect participants' action performance. So far, a direct and more detailed comparison of BAS and GAS actions' neural and behavioral processing is missing.

In the present fMRI study, we addressed the potential dissociation of BAS and GAS actions in the framework of predictive coding, suggesting that the brain works as a predictive device and is tuned to minimize its prediction errors (Friston, 2005). According to this model, action sounds are part of the predictive model that is engaged during action execution, or even the observation thereof (Friston, 2012). Neural responses in primary sensory cortices are attenuated for predicted self-generated sensations and the evidence is especially vast for self-initiated sounds (re-afferences, Baess et al., 2011; Kennel et al., 2015; Pizzera and Hohmann, 2015; Mifsud et al., 2016; Rummell et al., 2016; Timm et al., 2016), enabling the immediate registration of prediction-deviant sensations (prediction errors) and effective correction of sound-producing actions (Tourville et al., 2008). With regard to its neural underpinnings, prediction of self-produced sounds is considered to rely on a network consisting of the primary auditory cortex (Heschl's Gyrus) and the posterior superior temporal gyrus (pSTG; Rauschecker, 2012; Heilbron and Chait, 2018) and potentially also the supplementary motor area (SMA; Jo et al., 2019) and the cerebellum (Petrini et al., 2011; Waszak et al., 2012). The latter three structures are suggested to deliver a predictive sound model to primary auditory cortex, causing an attenuation of responses to expected sounds, and mismatch signals for unexpected sounds.

We examined potential differences between GAS and BAS as operationalized by tap dancing and hurdling, respectively. In both tap dancing and hurdling, the lower limbs are the effectors of audible sounds, and sounds produced by the lower limbs seem to show the same prediction-driven sensory attenuation as the more thoroughly examined sounds produced by our hands (van Elk et al., 2014). We trained naïve participants and filmed them during motor execution to create point-light displays with accompanying action sounds for both tap dancing and hurdling. During a subsequent fMRI session, the same participants were presented with the point-light videos of their own actions and asked to rate the subjective quality of their actions after each video on a 6-point Likert scale. To separately investigate the impact of visual and auditory information on BAS and GAS action processing, we additionally introduced different types of “scrambling” to the action videos serving as selective baseline conditions. Scrambling was applied to either the visual modality, the auditory modality, or both. While leaving the biological motion visually and audibly perceivable, scrambling strongly reduced the information about the quality of action performance. On the behavioral level, we expected overall lower rating scores for auditory scrambled videos, and due to the presumed greater importance of auditory feedback in GAS actions, we hypothesized that this effect would be especially pronounced in the tap-dancing condition.

Correspondingly, we expected tap dancing and hurdling to differ in their BOLD activity in auditory cortices reflecting that action sounds modulate GAS action processing more than BAS action processing. In particular, we expected activity in primary auditory cortex to be more attenuated for GAS as compared to BAS, based on the notion that effective sensory attenuation results from a prediction of sensory action effects (Wolpert et al., 1995; Miall and Wolpert, 1996; Friston et al., 2010; Schröger et al., 2015b). Furthermore, we reasoned that both GAS and BAS actions entail predictions about visuospatial motion patterns, whereas predictions about action sound patterns are pronounced for GAS actions. Regions sending a top-down signal to sensory cortices, especially SMA, the pSTG (Jo et al., 2019) and the cerebellum (Petrini et al., 2011; Waszak et al., 2012) should be more active in GAS than in BAS actions. Finally, prediction errors are suggested to travel up the predictive hierarchy to enable an adaptation of the current predictive model (Phillips et al., 2015; Heilbron and Chait, 2018). Therefore, we expected auditory scrambling to induce a predictive mismatch signal that manifests as increased BOLD response in the pSTG for GAS actions (Fu et al., 2006).

## Materials and Methods

### Participants

The original sample consisted of 19 participants. One participant left the study before finishing the 9-week training of hurdling and tap dancing. Therefore, video and audio data from 18 participants were processed further. Four participants dropped out of the study after the training, so that 14 participants took part in the fMRI session. One participant was excluded from the final analysis, because their reaction times recorded during the fMRI session diverged more than two standard deviations from the mean reaction time, leaving 13 participants (9 females, 4 males) for the analysis. While this is a relatively small sample size, it is comparable to other studies examining action sounds behaviorally (Menzer et al., 2010) or with fMRI (Reznik et al., 2015). The participants' age ranged from 19 to 28 years (*M* = 22.1, *SD* = 2.8), and all of them were right-handed, as assessed by the Edinburgh Handedness Inventory (EHI; Oldfield, 1971), scores varying from +60 to + 100, with a mean of +84. All participants reported to have no history of psychiatric or neurological disorders and signed an informed consent. After successful participation, participants were rewarded with both course credit and monetarily. The study was approved by the Local Ethics Committee of the University of Muenster (Department of Psychology) in accordance with the Declaration of Helsinki.

### Material

**Figure 1:**
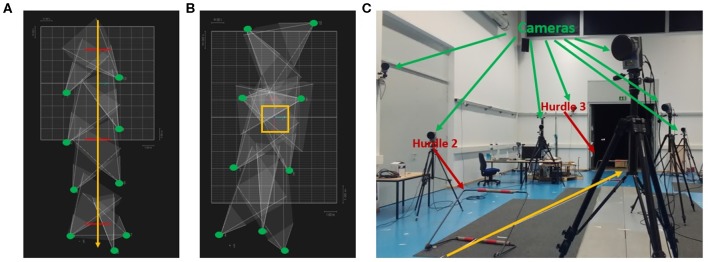
Camera positions and set-up during the point light recordings. **(A)** Camera positions during hurdling from a top view perspective. Green dots represent the cameras, red lines the hurdles, and the yellow arrow the hurdling track. **(B)** Camera positions during tap dancing from a top view perspective. Green dots represent the cameras, the yellow square the area in which the tap dancer performed the sequence. **(C)** Set-up during the recording of hurdling. Three hurdle transitions had to be performed during the recording. The two last hurdles are visible in the figure above. The yellow arrow indicates the hurdling track.

The stimuli consisted of point-light displays of hurdling and tap-dancing actions with the accompanying sounds, recorded from each participant individually at different stages during training. Point-light displays were recorded using the Qualisys Motion Capture System (https://www.qualisys.com) with nine cameras (see Figure 1), while the sound was recorded by in-ear microphones (Sound-man OKM Classic II) for hurdling and by a sound recording app on a mobile phone for tap dancing.

**Figure 2:**
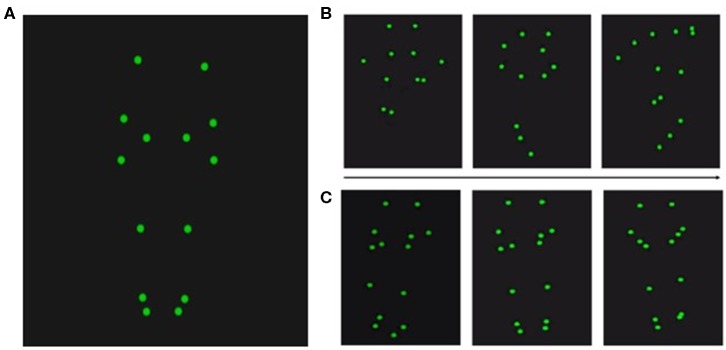
Position of the point-light markers. **(A)** Twelve point-light markers were used and positioned at the shoulders, elbows, wrists, hips, knees, ankles, and the tips of the toes. Exemplary videos can be found in Videos S1–S8. **(B)** Three snapshots of the hurdling action over the course of one video. **(C)** Three snapshots of the tap-dancing action over the course of one video.

After the acquisition, point-light displays were processed using Qualisys, ensuring visibility of all 12 recorded point-light markers during the entire recording time (for an overview of the position of the point-light markers, see Figure 2). Note that we selected only videos with error-free performance for our experiment, excluding BAS trials in which the hurdles were touched. Accordingly, all sounds in GAS and BAS were exclusively produced by foot-ground contacts.

Sound data were processed using Reaper v5.28 (Cockos Inc., New York, United States). In a first step, stimulus intensities of hurdling and tap-dancing recordings were normalized separately. In order to equalize the spectral distributions of both types of recordings, the frequency profiles of hurdling and tap-dancing sounds were then captured using the Reaper plugin Ozone 5 (iZotope Inc., Cambridge, United States). Finally, the difference curve (hurdling—tap-dancing) was used by the plugin's match function to adjust the tap-dancing spectrum to the hurdling reference (see Figure S1; examples of the sounds are given in Videos S1–S8). Point-light displays and sound were synchronized, and the subsequent videos were cut using Adobe Premiere Pro CC (Adobe Systems Software, Dublin, Ireland). The final videos had a size of 640 × 400 pixels, 25 frames per second, and an audio rate of 44 100 Hz. A visual fade-in and fade-out of 1 s (25 frames) were added with Adobe Premiere. Video length ranged from 3 to 6 s, with an average length of 5 s.

For the fMRI sessions, a subset of 27 hurdling and 27 tap dancing videos was selected for each participant, choosing the videos with the most reliable ratings from the test and retest sessions. For every selected video, additional “scrambled” versions were created using Adobe Premiere. The visual and auditory tracks of the videos were cut into 1-s segments (25 frames) and the segments were then rearranged. The same scrambling scheme was applied to all videos, that is, the segments were rearranged in a fixed order. We created three different types of “scrambling”- either the visual track, the auditory track, or both.

All videos were presented using the Presentation software (Version 18.1, Neurobehavioral Systems, Inc., Berkeley, CA).

### Procedure

#### Training and Filming Sessions

**Figure 3:**
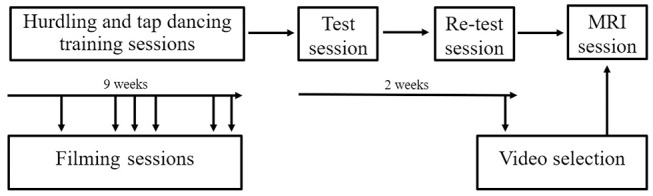
Procedure of the study. Participants were filmed on several occasions during their 9-week training in hurdling and tap dancing. Two behavioral sessions were conducted before the MRI session.

Participants engaged in a 9-week training period during which they were trained in hurdling and tap dancing by professional instructors (Figure 3). The training in both hurdling and tap dancing was conducted two times a week, with each training session having a length of 90 min, so that participants trained both action types for 3 h a week. Before this training, none of the participants ever practiced hurdling or tap dancing. During the 9-weeks training period, participants had to take part in four filming sessions, taking place at different states of training, to observe changes in performance. The first filming sessions took place 2 weeks after the training started, with the following filming sessions taking place in weeks four, five, six, eight, and nine after training commenced. Participants could choose four sessions from the provided ones. During the filming sessions, participants were equipped with 12 point-light markers (see Figure 2) and filmed via infra-red cameras of the motion capturing system while performing both action types. The hurdling action consisted of three hurdle transitions (Figure 1), while the tap-dancing action was a movement sequence learned in the tap-dancing training sessions. Both actions increased in difficulty with the four sessions. For hurdling, the spatial distance between the three hurdles increased, requiring more speed. Whereas, for tap dancing, action elements were added to the sequence to increase difficulty.

#### Behavioral Test and Retest Sessions

Behavioral test-retest sessions were conducted to find the videos with the highest reliability of participants' rating. Both sessions were conducted in a computer lab in the Department of Psychology at the University of Muenster. Participants were seated in front of a computer and instructed to rate the quality of their actions on a scale from 1 (“*not well at all*”) to 6 (“*very well*”) based on their subjective impression. The instructions were kept intentionally liberal as to not influence participants to favor specific aspects of the action for their evaluation. The experiment consisted of two blocks with self-paced responses, both lasting between 20 and 30 min. The same videos were presented in a different order in the second block of the experiment. Videos were pseudorandomized so that not more than three videos in a row showed the same action type (hurdling vs. tap dancing). Overall durations of the test session ranged from 40 to 60 min, depending on the participants' response speed. Two weeks after the test session, participants were presented the same videos once more (in pseudo-randomized order). Twenty-seven videos for both hurdling and tap dancing were chosen per participant and were used in the subsequent fMRI session. The videos with the highest reliability in rating were chosen. Every video was rated a total of four times (two times in the test and two times in the retest sessions). Of all chosen videos (702 videos in total, 54 per participant), 23.79% received the same rating on all four repetitions, in 69.8% ratings varied by a score of either +1 or −1 in one or two of the repetitions, and in 6.41% ratings varied by a score of ± 1.

#### fMRI Session

**Figure 4:**
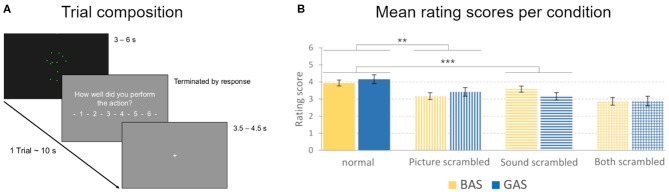
Trial composition and action quality rating scores. **(A)** A trial consisted of a video (3–6 s in length), followed by the video rating question (“How well did you perform the action?” in German), and a fixation cross (3.5–4.5 s in length). The total duration of one trial was approximately 10 s. **(B)** Mean rating scores for the evaluation of the quality of the action performance presented in the observed videos, obtained during the MRI sessions. Rating scores could range from 1 to 6 (1 representing a low, 6 a high rating of quality). Error bars show standard errors. BAS conditions are represented in yellow, GAS conditions in blue. Vertical stripes represent the scores for *picture-scrambled* conditions, whereas horizontal stripes represent *sound-scrambled* conditions. Columns with both vertical and horizontal stripes represent the conditions with both picture and sound scrambled. **p = 0.005, ***p = 0.001.

For the fMRI session, participants were instructed to rate the quality of their actions presented in the videos. They were informed that there would be “scrambled” videos, where visual and auditory input would not match, but they should still consider both modalities in the best way possible to rate the quality of their performance. Participants were asked to regulate the volume of the sounds before the experiment started to assure that the action sounds were audible above the scanning noises. The experiment consisted of nine blocks, including 28 trials each (Figure 4).

Transition probabilities ensured that every condition was preceded by every condition (including the same condition) in the same number of trials over the whole experiment. The first trial of a block was a repetition of the last trial of the preceding block, to avoid losing a transition. The remaining 27 trials consisted of 3 trials for each of the nine conditions. With the first trial after each pause discarded, 243 trials remained, 216 video trials, and 27 null events, where a fixation cross was presented (27 trials for each of the nine conditions). The duration of the null events was fixed at 5 s. Before every trial, a fixation cross was presented as an interstimulus interval, varying between 3.5 to 4.5 s in length. After every video trial, the six-point rating scale, including the rating question, was presented. The experiment continued upon the participants' button press.

After the experiment, lasting approximately 45 min, an 8-min resting-state sequence was acquired. Participants were asked to look at a fixation cross for the whole period. Throughout the entire scanning routine, participants were instructed to refrain from moving.

### fMRI Recordings and Preprocessing

Participants were scanned in a 3-Tesla Siemens Magnetom Prisma MR tomograph (Siemens, Erlangen, Germany) using a 20-channel head coil. A 3D-multiplanar rapidly acquired gradient-echo (MPRAGE) sequence was used to obtain high resolution T1 weighted images ahead of functional scanning, with scanning parameters set to 192 slices, a repetition time (TR) of 2,130 ms, an echo time (TE) of 2.28 ms, slice thickness of 1 mm, a field of view (FoV) of 256 × 256 mm^2^, and a flip angle of 8°.

Gradient-echo echoplanar imaging (EPI) was used to measure blood-oxygen-level-dependent (BOLD) contrast for functional imaging data of the whole brain. There were 10 EPI sequences in total. One sequence for the volume adjustment and one sequence for each of the nine experimental blocks. Scanning parameters were set to a TE of 30 ms, a TR of 2,000 ms, a flip angle of 90°, 33 slices with a slice thickness of 3 mm, and a FoV of 192 × 192 mm^2^.

Imaging data were processed using SPM12 (Wellcome Trust, London, England). Preprocessing consisted of slice time correction to the middle slice, realignment to the mean image, co-registration of the functional data to the individual's structural scan, normalization into the standard MNI space (Montreal Neurological Institute, Montreal, QC, Canada) based on segmentation parameters, and spatial smoothing with a Gaussian kernel of full-width at half maximum (FWHM) of 8 mm. A high-pass temporal filter equivalent to 128 s was applied to the data.

### Statistical Data Analysis

#### Behavioral Data Analysis

Firstly, we performed the Kolmogorov-Smirnov test to ensure the normal distribution of our rating scores. A 2 × 2 × 2 within-subject analysis of variance (ANOVA) was calculated to examine differences in the performance ratings between the eight experimental conditions, using SPSS (IBM, New York, United States). The first factor was action, with the factor levels *BAS (hurdling)* and *GAS (tap dancing)*, the second factor was picture with factor levels *picture normal* and *picture scrambled* and the third factor was sound with factor levels *sound normal* and *sound scrambled*.

We calculated *post-hoc t*-tests for significant main effects using a Bonferroni correction for multiple comparisons, which divides the significance threshold (here we use α = 0.05) by the number of tests (Bonferroni, 1936).

#### fMRI Design Specification

The design was implemented in SPM12, following a general linear model approach (GLM, Friston et al., 1994; Worsley and Friston, 1995). The modeled activation was time-locked to the onsets of the videos or null events. Epochs contained the full presentation period ranging from 3 to 6 s for the videos, and 5 s for the null events. Since tap dancing and hurdling differ with regard to their auditory event density, i.e., the number of distinguishable auditory sounds occurring per second, we controlled for this source of variance by introducing regressors of nuisance. To this end, we used the MIR Toolbox (Lartillot et al., 2008) to calculate the action sounds' event densities. The GLM consisted of 23 regressors in total: eight regressors for the experimental conditions, eight parametric regressors modeling the event densities for each of the eight experimental conditions, one regressor for the null events, and six regressors for the motion parameters (three translations and three rotations). Activation for 27 trials was considered for the modeling of each of the experimental regressors as well as for the null event regressor. All regressors were convolved with the hemodynamic response function.

On the first level, t-contrasts of the experimental conditions against null were calculated (condition > rest). These contrast images were then used to set up a flexible factorial design on the second level. The flexible factorial design was chosen because it accounts best for the within-subject factor. The model consisted of 21 regressors—eight regressors for the experimental conditions, and 13 regressors for the subject effects, one for each participant.

First, t-contrasts for the unscrambled conditions were calculated (BAS_normal > GAS_normal) to assess basic differences between tap dancing and hurdling precluding potential effects of visual scrambling on the use of auditory information. Resulting t-maps were corrected using false discovery rate (FDR) correction and a threshold of *p* < 0.05. Additionally, we defined F-contrasts for the main effect of action, thus including both the normal and the scrambled condition (BAS > GAS), and for the action × sound interaction effect. *T*-tests were calculated to examine the direction of effects. Region of interest (ROI) analyses were performed to test our anatomically specified hypotheses, using FDR-correction with a threshold of *p* < 0.001. Structural ROIs were defined using the automated anatomical labeling (AAL) atlas and created using the WFU PickAtlas toolbox (Maldjian et al., 2003) in SPM12. Firstly, we performed a ROI analysis for the primary auditory cortex (Heschl's gyrus) for the BAS>GAS contrast, to test for the hypothesized stronger sensory attenuation for tap dancing than for hurdling. Secondly, ROI analyses for the secondary auditory cortex (pSTG), the SMA, and cerebellum for the GAS>BAS contrast were performed, to investigate a stronger activation for tap dancing due to explicit sound predictions over and above visual predictions. Thirdly, we performed a ROI analysis for the action x sound interaction effect, using structural ROIs for the pSTG, to examine the differential involvement of secondary auditory cortices in the sound-scrambled versions of hurdling and tap dancing. We expected more activation in the tap-dancing condition due to a more pronounced mismatch whenever the sound did not fit the perceived action. We additionally extracted beta values from pSTG and the primary auditory cortex, to examine more subtle differences between the conditions.

## Results

### Behavioral Results

The rating scores for all conditions were normally distributed, as checked by a Kolmogorov-Smirnov test. For the analysis of the rating scores, a 2 × 2 × 2 within-subject ANOVA with the factors action (*BAS, GAS*), picture (*picture normal, picture scrambled*), and sound (*sound normal, sound scrambled*) was calculated. Our final sample size was *n* = 13. We calculated a *post-hoc* power analysis using GPower (Erdfelder et al., 1996), determining a critical *F*-value (defining the boundary for the rejection of the null hypothesis) of 4.75. All observed effects were well above this critical value, indicating that our findings were reliable even in this relatively small sample size.

First of all, we did not find a main effect for the factor action [*F*_(1, 12)_ = 0.009, *p* = 0.928], indicating that participants were not biased to rate either their hurdling or tap-dancing performance as superior. Importantly, this balanced rating provides a solid basis to interpret differences between BAS and GAS without a confounding bias by preference.

As hypothesized, there was a significant main effect for the factor sound [*F*_(1, 12)_ = 22.01, *p* < 0.001, η^2^ = 0.647], driven by lower rating for *sound scrambled* (*M* = 3.13, *SD* = 0.6, Figure 4B) than compared to *sound normal* (*M* = 3.67, *SD* = 0.53). Likewise, a significant main effect for the factor picture [*F*_(1, 12)_ = 11.86, *p* = 0.005, η^2^ = 0.497] was explained by higher rating scores in the *picture normal* (*M* = 3.71, *SD* = 0.53) vs. *picture scrambled* condition (*M* = 3.09, *SD* = 0.7, Figure 4B).

There was a significant action x sound interaction [*F*_(1, 12)_ = 11.67, *p* = 0.005, η^2^ = 0.493]. Paired *t*-tests revealed lower ratings for *sound scrambled* vs. *normal* for hurdling [*M* = 3.23, *SD* = 0.61 vs. *M* = 3.55, *SD* = 0.49; *t*_(12)_ = 2.91, *p* = 0.013] as well as for tap dancing [*M* = 3.03, *SD* = 0.82 vs. *M* = 3.79, *SD* = 0.81; *t*_(12)_ = 5.08, *p* < 0.001]. The three-way interaction between action, picture and sound reached significance [*F*_(1, 12)_ = 6.66, *p* = 0.024, η^2^ = 0.357; for *post-hoc t*-tests, see Table S1 and Table S2], corroborating that the impact of auditory scrambling was stronger on tap dancing than on hurdling.

We performed a post-experimental survey where we asked participants to rate on a 6-point Likert scale how difficult it was for them to evaluate hurdling and tap-dancing videos. The rating difficulty did not differ between hurdling (*M* = 3.77, *SD* = 1.17) and tap-dancing videos [*M* = 3.54, *SD* = 1.13, *t*_(12)_ = 0.507, *p* = 0.621].

Finally, as for the fMRI data, we aimed to control for potential confounds of the behavioral rating by stimulus event density. To this end, we tested whether the rating scores correlated with the event density of the corresponding condition (all *p* > 0.05), implementing the same 2 × 2 × 2 within-subject ANOVA for the event density values. Here, we did not find a significant action × sound interaction [action × sound, *F*_(1, 12)_ = 0.34, *p* = 0.573].

### fMRI results

**Figure 5:**
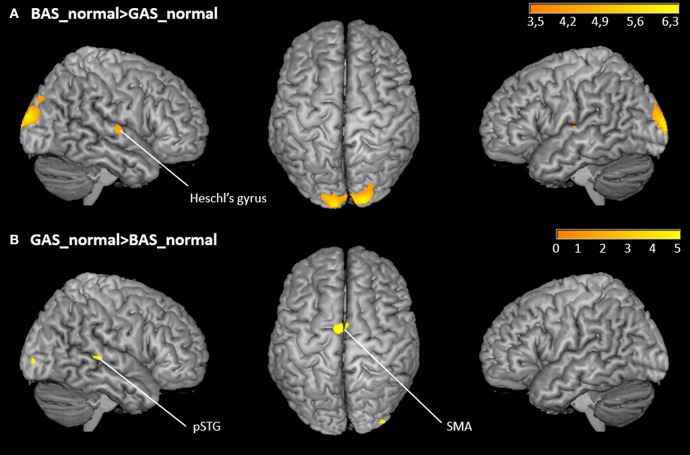
Whole-brain activation of the main effects of action. **(A)** FDR-corrected t-maps (*p* < 0.05) for the BAS_normal>GAS_normal contrast. **(B)** FDR-corrected t-maps (*p* < 0.05) for the GAS_normal>BAS_normal contrast.

The whole brain contrast BAS_normal>GAS_normal yielded higher activity in right primary auditory cortex (hypothesis 1) as well as in the occipital pole. Corroborating hypothesis 2, the reverse contrast GAS_normal>BAS_normal showed significant effects in SMA and right pSTG (Figure 5 and Table 1). Note that the same significant effects were observed when contrasting BAS and GAS aggregated for all their sub-conditions, i.e., effects were independent of auditory or visual scrambling.

**Table 1:** Whole-brain activation of the main effects of action.

	***x***	***y***	***z***	***t*-value**	**Voxels**
**BAS_normal>GAS_normal**					
Middle occipital gyrus	18	−100	20	6.45	718
Heschl's Gyrus	54	−13	5	4.70	39
	−51	−16	8	4.19	18
**GAS_normal>BAS_normal**					
SMA	−6	−7	65	5.51	42
pSTG	48	−34	5	4.92	30

*Regions of activation, MNI (Montreal Neurological Institute) coordinates (x, y, z), t-values for the local maxima (FDR-corrected, p <0.05), activation extent in voxels (clusters larger than k = 20)*.

The ROI analysis of the right and left Heschl's gyri yielded the expected lower activation of primary auditory cortex in GAS vs. BAS. According to the hypothesis of stronger sound prediction for the GAS>BAS contrast, the ROI analyses for the SMA, the cerebellum and the pSTG revealed also significant activation increases. ROI results are summarized in Table 2.

**Table 2:** Region of interest (ROI) results.

	***x***	***y***	***z***	***t*-value**	**Voxels**
**BAS>GAS**					
Heschl's Gyrus	54	−10	5	7.40	17
	−51	−16	8	5.99	5
**GAS>BAS**					
SMA	−3	−4	68	10.66	397
pSTG	54	−31	5	11.53	173
	−54	−31	23	6.72	131
	−54	5	−4	6.01	23
Cerebellum	−27	−58	−22	8.4	36
	24	−61	−19	7.95	62
	27	−64	−52	7.76	91
	−24	−64	−52	7.01	37

*MNI (Montreal Neurological Institute) coordinates (x, y, z), t-values for the local maxima (FDR-corrected, p <0.001), activation extent in voxels. ROI, region of interest; pSTG, posterior superior temporal gyrus; SMA, supplementary motor area*.

The ROI analysis for the interaction effect between action and sound, testing for a more pronounced prediction error in the auditorily scrambled GAS condition, did not show significant results in pSTG. Beta value extraction was performed to follow up on this, yielding a non-significant trend (*p* = 0.061) for the interaction effect (Figure S2). Additionally, we also extracted beta values from primary auditory cortex, to determine whether auditory scrambling of GAS would offset attenuation by increasing the prediction error. Indeed, we found a non-significant trend for the GAS_sound_scrambled>GAS_sound_normal comparison (*p* = 0.071).

## Discussion

Most of our actions generate sounds. Intuitively, these action sounds are important for controlling some of our actions, for instance speaking and singing, but possibly much less so for other types of action. The present study employed fMRI and an action quality rating task to investigate potential differences between actions that are executed in order to generate a particular action sound (GAS actions), and actions that cause sounds rather incidentally (BAS actions). Participants were presented with point-light videos showing themselves tap dancing (a GAS action) or hurdling (a BAS action). Following the predictive coding account, we hypothesized that the impact of predicted action sounds would be positively reflected in stronger activity of higher auditory areas (more auditory prediction) and correspondingly more pronounced attenuation in primary auditory cortex (less auditory mismatch). Moreover, trials where we introduced an experimental distortion of action sounds were expected to induce a stronger auditory prediction error in the respective network, and impair the quality rating more effectively, for GAS vs. BAS actions.

To determine the impact of visual and auditory information on the evaluation of one's own performance, we manipulated the visual and the auditory information by scrambling. As expected, both visual scrambling and auditory scrambling led to a significant reduction of the rating scores for hurdling and tap-dancing performance, with scores being the lowest when both modalities were scrambled at the same time. In line with our predictions, auditory scrambling had a stronger impact on the rating of tap-dancing than hurdling performance, with rating scores decreasing in both actions, but to a stronger degree in tap dancing. This finding supports the particular relevance of action sounds as an error-monitoring tool in GAS actions (Murgia et al., 2017). While auditory scrambling had an effect on the rating of the hurdling trials as well, the effect was more pronounced in tap dancing, showing that, with the auditory output being an explicit action goal, perception of action quality was especially reduced by incoherent auditory feedback. It is however important to note that action sounds were also important for the rating of hurdling performance, although the created sounds were not explicitly intended. Together, behavioral findings suggest that the brain generates predictions of how our actions should sound like, both in case of tap-dancing (GAS actions) as well as hurdling (BAS actions). Subtle differences in the level of interference, however, point to a more prominent role of auditory expectations in the former.

In agreement with our hypotheses, we found stronger activation in Heschl's gyri for the hurdling (BAS) compared to tap dancing (GAS) trials, reflecting a more pronounced sensory attenuation when auditory action consequences are predominantly used in the predictive model (GAS). Sensory attenuation to self-initiated sounds is based on an existing association between the initiated movement and the resulting sound (Ticini et al., 2012; Keysers and Gazzola, 2014). Self-produced sounds elicit a smaller amplitude in early EEG or MEG components, presumably due to the feeling of self-agency (Aliu et al., 2009; Baess et al., 2011; Timm et al., 2014). The precise origin of sensory attenuation of self-produced sounds has not yet been completely unraveled (Hughes et al., 2013; Horváth, 2015). A more recent study shows that sensory attenuation is not solely due to the self-generation of action sounds, but relies on the predictability of sensory input (Kaiser and Schütz-Bosbach, 2018). Self-generated sounds have high predictability, as the sensory effects are part of our motor plan when initiating and performing a movement (Shin et al., 2010). Accordingly, we suppose that tap-dancing sounds, being an intentional part of our motor plan, are more efficiently attenuated by internal predictive models, as reflected by stronger pSTG and SMA activity. In contrast, hurdling sounds may be less relevant in the predictive model, and are therefore not attenuated to the same extend in primary auditory cortices as the tap-dancing sounds.

Notably, attention was found to reverse attenuation effects by predicted stimuli, leading to enhanced rather than attenuated responses (Reznik et al., 2015; Schröger et al., 2015a,b; Wollman and Morillon, 2018). If at all, we would have expected attention to be increased for GAS vs. BAS actions. To the contrary, primary auditory cortex was attenuated in GAS compared to BAS, clearly favoring the prediction-caused attenuation over the attention-caused enhancement explanation of our findings. Corroborating this interpretation further, sound scrambling in GAS videos caused an increase of primary auditory cortex activity (non-significant trend), as would be expected for a prediction error rather than for a down-regulation of attention to the scrambled signal.

As expected, the SMA, the pSTG and the cerebellum were more active for tap-dancing than for hurdling. These and adjacent areas have been found and discussed in connection with action sound processing more generally (Herrington et al., 2011; Bischoff et al., 2014; Woods et al., 2014; Reznik et al., 2015). Reznik et al. (2015) proposed that predictive information is sent from the SMA or primary motor cortex to auditory cortices to modify activation during active sound generation. Although our participants did not actively create sounds in the scanner, they perceived sounds they actively created in the past, and perception of own past actions is thought to adequately represent the brain activity during action execution (e.g., Sato, 2008; Wutte et al., 2012). The same areas as found by Reznik et al. (2015) were more active in our tap-dancing condition compared to hurdling, indicating a similar predictive information update when action sounds are part of the intended action goal (GAS vs. BAS). This matches the stronger—less attenuated—effect in primary auditory cortex for hurdling trials as well.

While BOLD contrasts did not confirm the differential effect of auditory scrambling on tap-dancing and hurdling, beta estimates extracted from the pSTG did indicate a descriptive interaction effect for the imaging data. Thus, the beta weights for hurdling did not differ between the *sound normal* and *sound scrambled* condition, whereas there was a small difference between these two conditions for tap dancing. Both these findings and the behavioral results speak in favor of our interaction hypothesis: While auditory scrambling has an effect on both hurdling and tap dancing, the effect on tap dancing is larger, indicating a greater relevance of the auditory domain to positively evaluate action quality and a stronger predictive mismatch in GAS actions.

The overall stronger activation in occipital visual areas for hurdling compared to tap-dancing was not hypothesized. Obviously, evaluating one's hurdling performance yielded a more extensive visual processing of the observed action, as indicated by increased BOLD activity in occipito-temporal cortices (Jastorff et al., 2010). This also matches the behavioral finding of a stronger impact of visual scrambling on the rating of hurdling quality.

Overall, our behavioral and fMRI findings speak in favor of a higher relevance of action sounds in tap dancing as compared to hurdling. These actions may be representative for two subclasses of sound-producing actions, but their distinction might reflect two manifestations on a continuum rather than a strict dichotomy. As we observed, the auditory scrambling reduced rating scores in hurdling trials as well, indicating that the auditory domain is not completely unnecessary when evaluating these actions. This aligns with several previous findings regarding the relevance of sound when performing and improving sport related actions (for a review, see Schaffert et al., 2019). Also, effects of deprivation or alteration of auditory feedback on musical performance is not completely consistent, with some studies showing no effect of at least deprived feedback (Gates et al., 1974; Finney, 1997). A continuum, reaching from language production, where the auditory output is inarguably important, to simple everyday actions producing sounds, like placing a glass on a table, seems plausible. Our chosen actions might be somewhere in between, with tap dancing being closer to language and music, and hurdling closer to simple everyday action sounds. Note that this difference is particularly remarkable given that hurdling and tap-dancing are both whole-body actions and produce sounds by feet-floor contact, ruling out confounding impact on the motor side.

Future studies should avoid differences in event density as a potential source of confounding variance. While we controlled for this factor in both the fMRI and the behavioral analysis, event density could have been limited right from the beginning by choosing a tap-dancing sequence that largely matches the rhythm generated by the hurdling movement.

A limitation of our study is the small number of participants (*n* = 13), which resulted from the large extent of the investigation, including a 9-week training, several filming sessions, as well as multiple experimental sessions, both behavioral and fMRI. However, other studies examining action sound had a comparably small sample size (Menzer et al., 2010; Reznik et al., 2015). Considering that we found both robust behavioral interaction effects and FDR-corrected imaging results despite the limited number of participants, speaks in favor of a further pursue of our hypotheses with a larger sample size. An interesting approach might be to use the same stimuli with naïve participants who have not trained hurdling and tap dancing before, generalizing the results to participants who are no experts of the performed actions.

To further clarify the role of action sounds for monitoring action performance, future studies may examine the effects of deprivation and interference on the entire spectrum of sound-producing actions, as has been done for language production, musical performance, and some other actions (Howell, 2004; Keough and Jones, 2009; Pfordresher and Beasley, 2014; Kennel et al., 2015). Our study is a first step into a more systematic approach to understanding action sounds, while establishing ideas for additional research to deepen the comprehension of this relevant topic. Both musicians and athletes might benefit from a better understanding of the role of action sounds for optimizing action performance, giving them the opportunity to adequately train their skills. Research in schizophrenia might also gain from a better insight into the connections between action sounds and motor control, especially regarding the sense of agency, and the failure to attribute self-produced sensations to oneself in people with schizophrenia.

## Conclusion

In conclusion, our study provides interesting new insights on action sounds and their relevance for evaluating executed actions. In contrast to other studies, we trained our participants in two sound-producing actions and showed them their own actions during an fMRI experiment. This is, to our knowledge, completely novel in this field of research and thus provides a unique view on how our own action sounds are processed in the brain, depending on whether sound is an intentional action goal (tap dancing) or is generated incidentally (hurdling). Our results indicate that in the former case, the brain intensifies auditory predictions, and is more surprised in case of unexpected action sounds; moreover, these are particularly harmful to quality rating on a behavioral level. Research on real-life and whole-body action sounds is still relatively sparse, although they are omnipresent in our everyday life and supposedly important for controlling, understanding, and improving at least some of our actions. Finding that goal-relevance on a subjective level modulates brain processes during sound appraisal points out that this field of research is worth further exploration.

## Data Availability Statement

The datasets generated for this study are available on request to the corresponding author.

## Ethics Statement

The studies involving human participants were reviewed and approved by Ethics Committee Department of Psychology University of Muenster. The patients/participants provided their written informed consent to participate in this study.

## Author Contributions

NH, JP, DK, and RS contributed conception and design of the study. NH performed the statistical analysis and wrote the first draft of the manuscript. IT and RS wrote sections of the manuscript. KZ, MR, and RS contributed with scientific support, supervision, and coordination. All authors contributed to manuscript revision, read, and approved the submitted version.

## Conflict of Interest

The authors declare that the research was conducted in the absence of any commercial or financial relationships that could be construed as a potential conflict of interest.
