# Supplementary material for: Incidental or Intentional? Different Brain Responses to One's Own Action Sounds in Hurdling vs. Tap Dancing
Source: Front Neurosci. 2020 May 13;14:483. doi: 10.3389/fnins.2020.00483 (PMC7237737; doi:10.3389/fnins.2020.00483)
Supplement: Supplementary file 9 [file Data_Sheet_1.docx]

**
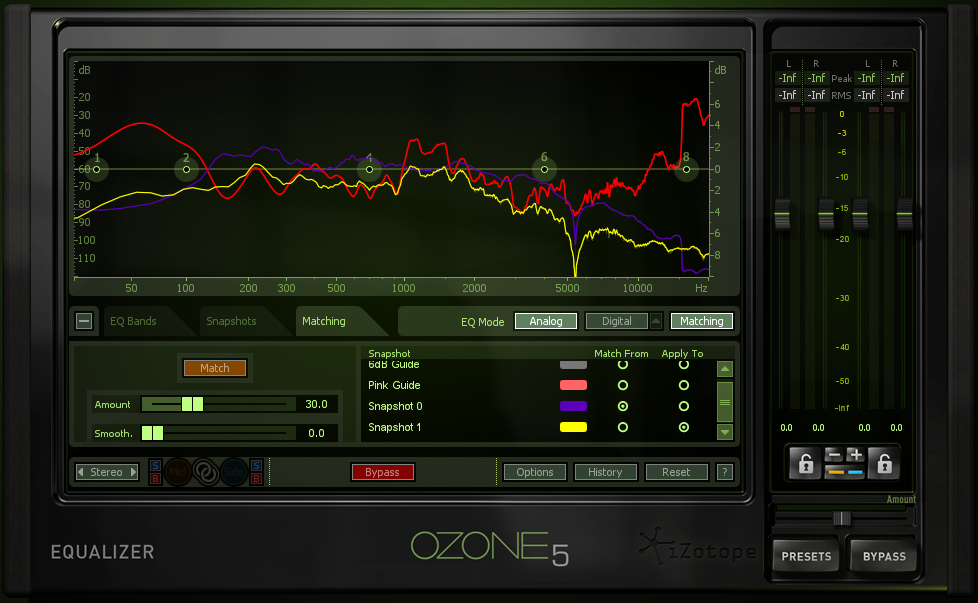
**

**Fig. S1. Matching of frequency spectra.** After intensity normalization, we used Ozone 5’s frequency matching function to capture frequency distributions of hurdling (yellow curve) and tap-dancing recordings (purple). Using hurdling as a reference, the difference curve (hurdling – tap-dancing, red) was computed to adjust the frequency distribution of tap-dancing to match those of hurdling recordings. The amount of frequency matching (see ‘Amount’ slider, bottom left) was set to make sure that no clipping would occur after applying the equalization. Note that the smoothing parameter was set to zero, ensuring a close approximation of both distributions.
