# Supplementary material for: Incidental or Intentional? Different Brain Responses to One's Own Action Sounds in Hurdling vs. Tap Dancing
Source: Front Neurosci. 2020 May 13;14:483. doi: 10.3389/fnins.2020.00483 (PMC7237737; doi:10.3389/fnins.2020.00483)
Supplement: Supplementary file 10 [file Data_Sheet_2.docx]

**
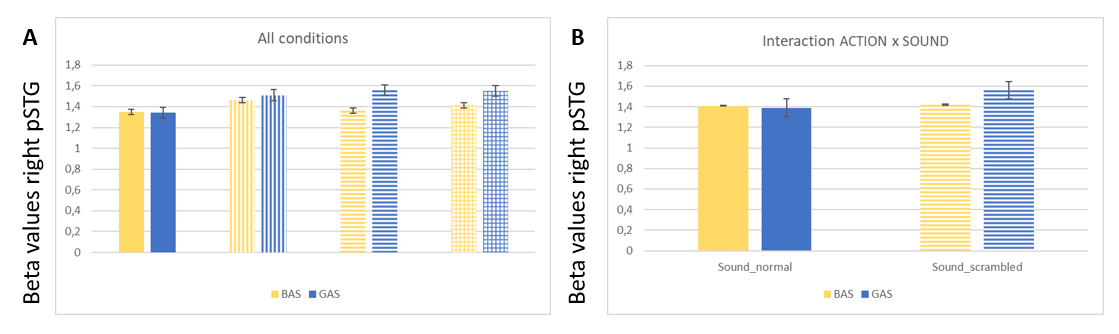
Fig. S2. Beta weights within the superior temporal gyrus.** Mean beta weights extracted from the right posterior superior temporal gyrus (A) for all eight experimental conditions and (B) divided by the factors action and sound to represent the interaction effect. Error bars represent the standard error of the mean. BAS conditions are represented in yellow, GAS conditions in blue. Vertical stripes represent the picture-scrambled conditions, whereas horizontal stripes represent sound-scrambled conditions. Columns with both vertical and horizontal stripes represent the conditions with both picture and sound scrambled.
