# Supplementary material for: Incidental or Intentional? Different Brain Responses to One's Own Action Sounds in Hurdling vs. Tap Dancing
Source: Front Neurosci. 2020 May 13;14:483. doi: 10.3389/fnins.2020.00483 (PMC7237737; doi:10.3389/fnins.2020.00483)
Supplement: Supplementary file 11 [file Data_Sheet_3.docx]

**Table S1 – Post-hoc t-tests for the two-way interactions**

| Interaction effect action x picture | | | | |
| --- | --- | --- | --- | --- |
|  | *M* | *SD* | *t* | *p* |
| BAS picture normal | 3.76 | 0.56 | 3.59 | .004* |
| BAS picture scrambled | 3.02 | 0.71 |  |  |
| GAS picture normal | 3.67 | 0.75 | 2.97 | .012* |
| GAS picture scrambled | 3.15 | 0.90 |  |  |
| BAS picture normal | 3.76 | 0.56 | 0.43 | .676 |
| GAS picture normal | 3.67 | 0.75 |  |  |
| BAS picture scrambled | 3.02 | 0.71 | 0.59 | .564 |
| GAS picture scrambled | 3.15 | 0.90 |  |  |
| Interaction effect action x sound | | | | |
|  | *M* | *SD* | *t* | *p* |
| BAS sound normal | 3.55 | 0.49 | 2.91 | .013* |
| BAS sound scrambled | 3.23 | 0.61 |  |  |
| GAS sound normal | 3.79 | 0.81 | 5.08 | < .001* |
| GAS sound scrambled | 3.03 | 0.82 |  |  |
| BAS sound normal | 3.55 | 0.49 | 1.06 | .308 |
| GAS sound normal | 3.79 | 0.81 |  |  |
| BAS sound scrambled | 3.23 | 0.61 | 0.89 | .392 |
| GAS sound scrambled | 3.03 | 0.82 |  |  |

BAS = by-product action sounds, GAS = goal-related action sounds*, M* = mean, *SD* = standard deviation, significant differences are marked with asterisks (*). Values are Bonferroni-corrected for multiple comparisons.

**Table S2 – Post-hoc t-tests for the three-way interactions**

|  |  | Interaction effect action x picture x sound | | | | | | |
| --- | --- | --- | --- | --- | --- | --- | --- | --- |
|  | | |  |  | *M* | *SD* | *t* | *p* |
| BAS | | | Picture normal | Sound normal | 3.93 | 0.6 | 2.57 | .024* |
|  | | |  | Sound scrambled | 3.58 | 0.61 |  |  |
|  | | | Picture scrambled | Sound normal | 3.17 | 0.70 | 2.54 | .026* |
|  | | |  | Sound scrambled | 2.87 | 0.78 |  |  |
| GAS | | | Picture normal | Sound normal | 4.16 | 0.90 | 5.01 | < .001* |
|  | | |  | Sound scrambled | 3.17 | 0.75 |  |  |
|  | | | Picture scrambled | Sound normal | 3.42 | 0.87 | 4.38 | .001* |
|  | | |  | Sound scrambled | 2.88 | 0.99 |  |  |
|  | | |  |  | *M* | *SD* | *t* | *p* |
| Picture normal | | | Sound normal | BAS | 3.93 | 0.60 | -0.91 | .382 |
|  | | |  | GAS | 4.16 | 0.90 |  |  |
|  | | | Sound scrambled | BAS | 3.58 | 0.61 | 1.92 | .080 |
|  | | |  | GAS | 3.17 | 0.75 |  |  |
| Picture scrambled | | | Sound normal | BAS | 3.17 | 0.70 | -1.14 | .278 |
|  | | |  | GAS | 3.42 | 0.87 |  |  |
|  | | | Sound scrambled | BAS | 2.87 | 0.78 | -0.07 | 0.946 |
|  | | |  | GAS | 2.88 | 0.99 |  |  |
|  | | |  |  | *M* | *SD* | *t* | *P* |
| BAS | | | Sound normal | Picture normal | 3.93 | 0.6 | 3.16 | .008* |
|  | | |  | Picture scrambled | 3.17 | 0.70 |  |  |
|  | | | Sound scrambled | Picture normal | 3.58 | 0.61 | 3.86 | .002* |
|  | | |  | Picture scrambled | 2.87 | 0.78 |  |  |
| GAS | | | Sound normal | Picture normal | 4.16 | 0.90 | 3.74 | .003* |
|  | | |  | Picture scrambled | 3.42 | 0.87 |  |  |
|  | | | Sound scrambled | Picture normal | 3.17 | 0.75 | 1.66 | .123 |
|  | | |  | Picture scrambled | 2.88 | 0.99 |  |  |

BAS = by-product action sounds, GAS = goal-related action sounds*, M* = mean, *SD* = standard deviation, significant differences are marked with asterisks (*). Values are Bonferroni-corrected for multiple comparisons.
