# Supplementary material for: Incidental or Intentional? Different Brain Responses to One's Own Action Sounds in Hurdling vs. Tap Dancing
Source: Front Neurosci. 2020 May 13;14:483. doi: 10.3389/fnins.2020.00483 (PMC7237737; doi:10.3389/fnins.2020.00483)
Supplement: Supplementary file 12 [file Data_Sheet_4.docx]

**Video S1. Example video for the BAS normal condition.**

**Video S2. Example video for the BAS picture scrambled condition.**

**Video S3. Example video for the BAS sound scrambled condition.**

**Video S4. Example video for the BAS picture sound scrambled condition.**

**Video S5. Example video for the GAS normal condition.**

**Video S6. Example video for the GAS picture scrambled condition.**

**Video S7. Example video for the GAS sound scrambled condition.**

**Video S8. Example video for the GAS picture sound scrambled condition.**
